# Nurse-led telephone follow-up according to the revised nursing outcomes classification for laryngeal carcinoma surgery patients: a randomized controlled trial

**DOI:** 10.1186/s12912-022-01054-2

**Published:** 2022-10-17

**Authors:** Yongxia Ding, Jinxia Xu, Yan Ning, Qian Wang, Zhaojie Chang

**Affiliations:** 1 Nursing College of Shanxi Medical University, No. 98, University Street, 030600 Jinzhong, China; 2 Shanxi Key Laboratory of Otolaryngology, Head and Neck Cancer, NO.85 Jiefang South Road, 030001 Taiyuan, China; 3 Shanxi Cancer Hospital, No.3 Xincun Street, 030013 Taiyuan, China

**Keywords:** Nursing outcomes, Telephone follow-up, Laryngeal carcinoma surgery, Quality of life

## Abstract

**Background:**

This study used the revised nursing outcomes classification (NOC) outlined in our previous study, “Core nursing outcomes for otorhinolaryngology head-neck,” for telephone follow-up of patients who had laryngeal carcinoma surgery in China. This randomized controlled trial aimed to compare nurse-led telephone follow-up according to the revised NOC with traditional telephone follow-up.

**Methods:**

A total of 100 postoperative patients were recruited from March 2018‒March 2020. Patients were randomly assigned to nurse-led telephone follow-up groups as either revised NOC follow-up (n = 51) or traditional follow-up (n = 49). The investigated outcomes included nursing outcomes, quality of life, and self-care abilities.

**Results:**

The baseline characteristics of patients were well balanced. We noted that improvements in nursing outcomes in the intervention group were significantly better than for those in the control group (*P* = 0.018), primarily regarding psychosocial health (*P* < 0.001) and health knowledge and behavior (*P* < 0.001). Moreover, patient outcomes in the intervention group were associated with greater improvements in quality of life than those in the control group (*P* < 0.001), especially for social conditions (*P* < 0.001), emotional health (*P* < 0.001), functional status (*P* < 0.001), and additional attention (*P* = 0.001). Finally, compared with the control group, significant improvements were observed in self-care abilities in the intervention group (*P* = 0.002), mainly regarding general self-care abilities (*P* = 0.016) and development self-care abilities (*P* < 0.001).

**Conclusion:**

This study found that nurse-led telephone follow-up according to the revised NOC improved nursing outcomes, quality of life, and self-care abilities.

**Trial registration:**

ChiCTR2100045941.

**Supplementary Information:**

The online version contains supplementary material available at 10.1186/s12912-022-01054-2.

## Background

Laryngeal carcinomas are the most common type of malignant tumors that receive treatment in otorhinolaryngology departments [1]. The prevalence of laryngeal carcinoma is increasing; it currently accounts for 1‒5% of all malignant tumors and 11‒22% of head and neck malignant tumors [2, 3]. Further, tumor size, local invasion, spread into lymph nodes, and distant metastasis can affect the prognosis for laryngeal carcinoma [4]. Chemoradiotherapy is used to treat laryngeal carcinoma in its early stage, whereas surgery is widely used in its advanced stage, including total and partial laryngectomies [5, 6]. Patients who undergo a laryngectomy may have a long-term need for a laryngeal tube, and the resultant loss of speech can affect quality of life and self-care abilities [7, 8].

After surgery, laryngeal carcinoma patients may experience physical and mental health problems. The guidance they receive should be normative and professional. Nurse-led telephone follow-up is the most frequently used approach for providing follow-up care that includes individual health guidance and psychological support. Telephone follow-up has already been introduced for patients in various settings, including follow-up after urological surgery, cataract surgery, Mohs micrographic surgery, pelvic floor surgery, and for patients experiencing obstructive sleep apnea [9–13]. Research has shown that health care quality and the burden of postoperative symptoms or patient satisfaction could improve if patients receive telephone follow-up [9–13]. However, telephone follow up may be too short in duration, and the effectiveness of the delivered content may depend on the nurse’s communication skills. The Nursing Outcomes Classification (NOC) contains 490 and 540 core nursing outcomes in the 5th Edition and 6th Edition, respectively, that assess patients’ responses to interventions and the patient follow-up process [14, 15]. Our previous study identified suitable postoperative nursing outcomes for laryngeal carcinoma in China, including 14 core postoperative nursing outcomes and 69 indicators [16]. However, it is unclear whether utilizing this revised NOC to structure nurse-led telephone follow-up is more effective than traditional telephone follow-up. Therefore, this study compared nurse-led telephone follow-up that was structured according to the revised NOC with traditional telephone follow-up for laryngeal carcinoma surgery patients. The primary outcome was to improve nursing outcomes, and the secondary endpoints were quality of life and self-care abilities.

## Methods

### Trial Design and Oversight

The study was a randomized, observer-blinded, two-armed, single-center trial, and all patients were recruited from the Shanxi Key Laboratory of Otolaryngology, Head and Neck Cancer, China. The ethics committee of the Nursing College of Shanxi Medical University (2020sll002) approved the protocol and amendments, and the study’s sponsor (Yongxia Ding) was responsible for data collection. Moreover, every enrolled patient signed an informed written consent form. Patients were randomly assigned to nurse-led telephone follow-up according to the revised NOC (intervention group) or traditional telephone follow-up (control group), with a 1:1 ratio, using a permuted block randomization method. A statistician generated random allocation sequence, and a research assistant enrolled and assigned patients. The details of the revised NOC were reported in our previous study, which contained 14 core postoperative nursing outcomes and 69 indicators [16]. These items were subdivided into physiological health, psychosocial health, health knowledge and behavior, and perceived health; details of this study can be found in Supplementary file 1. Patients completed a questionnaire during their follow-up periods, and follow-up assessments were conducted on both groups six months post the study period [17].

### Study participants

One hundred patients who had undergone laryngectomy were recruited from March 2018‒March 2020. The following inclusion criteria were applied: (1) laryngeal carcinoma confirmed by pathology, (2) treated with total or partial laryngectomy, and (3) patient understands the questionnaire content. Patients with the following characteristics were excluded: (1) recurrence or metastasis of laryngeal carcinoma within six months; (2) cancer at other sites; (3) serious heart, brain, or kidney disorders; (4) mental health issues under treatment; (5) restricted communication ability in terms of speaking, listening, reading, writing, or other forms of communication; and (6) the primary caregiver works in health care.

### Data Collection and Endpoints Assessment

The general characteristics of patients were obtained from uniform medical records; the information included the patients’ sex, age, educational status, residential location, occupation, marital status, income, smoking history, alcohol history, surgical approach, tube use, and radiotherapy status. The primary endpoint was core nursing outcome scores assessed by the nurse based on physiological health, psychosocial health, health knowledge and behavior, and perceived health [16]; secondary endpoints were quality of life and self-care abilities.

Quality of life after laryngectomy was assessed using the Chinese version of the Functional Assessment of Cancer Therapy–Head and Neck, which was devised by the Center for Translational Research and Education at Northwestern University, USA [18]. The Chinese version of the Functional Assessment of Cancer Therapy–Head and Neck has 5 dimensions and 38 hierarchical items. Each item is scored from 0 to 4, with a total range of 0‒152; a high score for life quality indicates a better quality of life (Supplementary file 2). Self-care ability was assessed using the Chinese version of the Appraisal of Self-Care Agency Scale, which comprises the domains of general self-care skills, developed self-care skills, and self-care skills with poor health status [19]. General self-care skills comprise six items covering a patient’s abilities to maintain the normal and functional integrity of the self. Developed self-care skills are those abilities that are needed under specific conditions and comprise four items. Self-care skills with poor health status comprise five items needed by a patient with a disease or medical issue. Each item is scored from 1 to 5, and high scores indicate better self-care abilities (Supplementary file 3). All of the investigated outcomes were collected before intervention, and at 6 months after discharge through patients hospital revisit. The investigator was trained by the research group to master the concept and evaluation method. The general characteristics questionnaire, quality of life questionnaire, and self-care ability questionnaire were distributed to the patients on the spot and completed by the patients independently. In case participants did not understand the question, the question was explained without being prompted about how to respond. When it was inconvenient for the patient to fill out the form, the investigator would read the item out to the patient, and complete the questionnaire based on the patient’s answer.

### Intervention and control groups

Telephone follow-ups were conducted by four specialist nurses, two nursing management graduate students, and two otolaryngology head and neck surgery graduate students assigned to the intervention and control groups. The specialist nurses were registered nurses specializing in otolaryngology head and neck surgery with (1) at least two years of experience, (2) clinical experience with laryngeal carcinoma, (3) an undergraduate college degree or above, and (4) approval to participate in the study and complete the follow-up tasks in the allotted time. Nurses in the intervention group were trained in motivational communication according to the revised NOC [16]. All of the nurses received three training sessions and the duration of each session was 1 h. The follow-up sequence was connect, introduce, communicate, ask and respond, and exit. (1) *Connect* consists of politely confirming if the person answering the phone is the patient or a family member. (2) The nurse *introduces* herself and her role and states the purpose of the follow-up. (3) In *communicate*, the nurse obtains the patient’s consent and cooperation. (4) In *ask and respond*, the patient is asked the questions from the revised NOC, and the nurse provides health guidance for the patient. (5) *Exit* consists of the nurse thanking the patient for their cooperation and making an appointment for the next follow-up call.

The details for the questions for the telephone follow-up conducted according to the revised NOC are shown in Supplementary file 4. The traditional telephone follow-up focused on postoperative recovery, answered the questions raised by patients, and provided health guidance without unified and standardized follow-up content. Patients were followed-up at one, two, four, six, and eight weeks after discharge; thereafter, a follow-up call was made every eight weeks, which was consistent with the National Comprehensive Cancer Network and the requirements of follow-up postoperative care [20]. A total of seven calls were made to patients in the intervention and control groups.

### Sample size

The sample size was calculated based on the trial conducted by Malmstrom et al. [21]; 41 patients were required for each group, with α = 0.05 and β = 0.20. Considering a dropout rate of 10%, the sample size needed was higher than 91. Hence, the total sample size in our study was 100 patients. The sample size in this study was calculated with the PASS software, version 11.

### Statistical analysis

The investigated outcomes were assessed before discharge and six months after discharge. Furthermore, categorical data were described by event (proportion), whereas continuous data were shown as mean (standard deviation) or median (quartile) based on whether the data met a normal or abnormal distribution, respectively. The chi-square test was applied to assess the differences in baseline characteristics between the intervention and control groups owing to all variables being categorical data. As the data for nursing outcomes, quality of life, and self-care abilities met a normal distribution, comparisons of these variables before and after intervention in each group were assessed using paired *t*-tests. Moreover, the mean changes in nursing outcomes, quality of life, and self-care abilities between groups were evaluated using independent *t*-tests. All *P* values were two-sided, and *P* < 0.05 is considered statistically significant. IBM SPSS 19.0 was applied for statistical analysis.

## Results

### Baseline characteristics

**Fig. 1:**
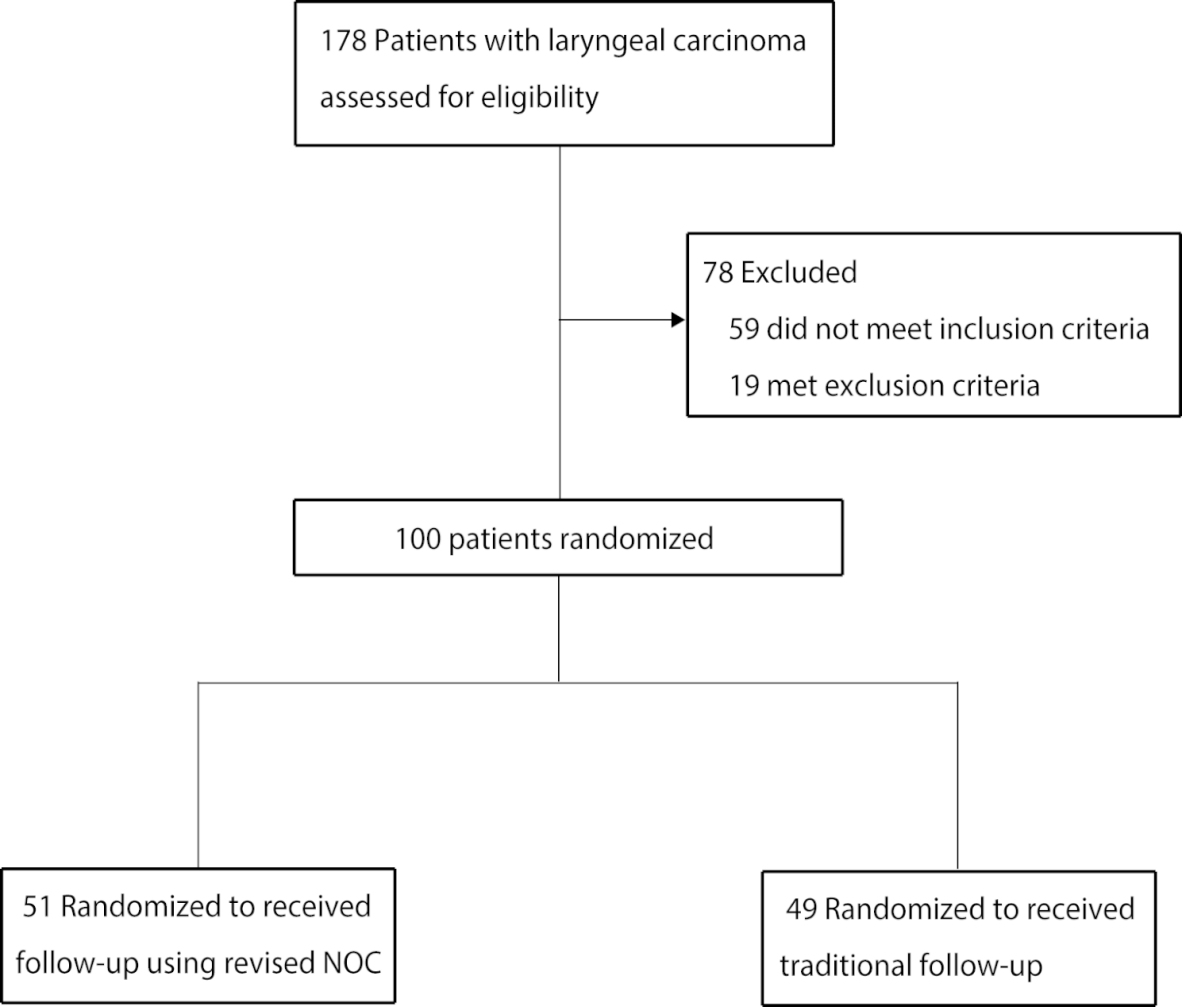
Flowchart of the study population

Of the 178 patients who underwent laryngectomy between March 2018 and March 2020 at the participating recruitment center, 100 were randomized: 51 to the intervention group and 49 to the control group (Fig. 1). The baseline characteristics of the recruited patients are shown in Table 1. There were no statically significant differences between groups for sex, age, educational status, residential location, occupation, marital status, income, smoking, alcohol, surgical approach, with tube, and radiotherapy status.

**Table 1:** Baseline Characteristics of Included Patients

Variable	Group	Intervention (n = 51)	Control (n = 49)	***P*** value
Sex	Male	45 (88.2)	43 (87.8)	0.941
	Female	6 (11.8)	6 (12.2)	
Age (years)	< 50.0	7 (13.7)	6 (12.2)	0.975
	50.0–70.0	37 (72.6)	36 (73.5)	
	> 70.0	7 (13.7)	7 (14.3)	
Educational status	Primary schools	8 (15.7)	7 (14.3)	0.990
	Junior high school	25 (49.0)	24 (49.0)	
	High school	12 (23.5)	12 (24.5)	
	College degree or above	6 (11.8)	6 (12.2)	
Residential location	Village	28 (54.9)	24 (49.0)	0.553
	Cities and towns	23 (45.1)	25 (51.0)	
Occupation	Farmer	28 (54.9)	26 (53.1)	0.929
	Worker	12 (23.5)	14 (28.6)	
	Public official	3 (5.9)	2 (4.1)	
	Other	8 (15.7)	7 (14.3)	
Marital status	Married	48 (94.1)	48 (98.0)	0.317
	Widowed	3 (5.9)	1 (2.0)	
Income	Low (< 5000 Yuan)	26 (51.0)	21 (42.9)	0.563
	Moderate (5000–10,000 Yuan)	19 (37.3)	20 (40.8)	
	High (> 10,000 Yuan)	6 (11.8)	8 (16.3)	
Smoking	No	7 (13.7)	4 (8.2)	0.374
	Yes	44 (86.3)	45 (91.8)	
Alcohol	No	12 (23.5)	11 (22.4)	0.898
	Yes	39 (76.5)	38 (77.6)	
Surgical approach	Total laryngectomy	14 (27.5)	12 (24.5)	0.736
	Partial laryngectomy	37 (72.5)	37 (75.5)	
With tube	Yes	29 (56.9)	27 (55.1)	0.859
	No	22 (43.1)	22 (44.9)	
Radiotherapy	Yes	35 (68.6)	36 (73.5)	0.594
	No	16 (31.4)	13 (26.5)	

### Nursing outcomes

The results of the core nursing outcome scores are shown in Table 2. After intervention, the items for physiological health, psychosocial health, health knowledge and behavior, perceived health, and total scores were significantly higher than those before intervention in both groups (*P* < 0.001). Moreover, patients in the intervention group experienced greater improvements in psychosocial health (9.34 ± 2.78 vs. 4.80 ± 3.09; *P* < 0.001), health knowledge and behavior (12.52 ± 9.27 vs. 5.94 ± 8.12; *P* < 0.001), and total scores (67.10 ± 25.52 vs. 53.36 ± 31.24; *P* = 0.018) than those in the control group. However, differences in physiological health (39.08 ± 15.96 vs. 36.70 ± 22.55; *P* = 0.554) and perceived health (6.16 ± 1.91 vs. 5.92 ± 2.11; *P* = 0.552) between the intervention and control groups were not statistically significant.

**Table 2:** Comparison of Core Nursing Outcomes Scores

Items	Before intervention	After intervention	Mean difference	***P*** value for mean change	***P*** value between group
Physiologic Health					0.544
Intervention group (n = 51)	125.96(27.28)	165.04(13.46)	39.08(15.96)	< 0.001	
Control group (n = 49)	125.76(28.47)	162.46(17.50)	36.70(22.55)	< 0.001	
Psychosocial Health					< 0.001
Intervention group (n = 51)	22.48(3.88)	31.82(2.40)	9.34(2.78)	< 0.001	
Control group (n = 49)	22.50(3.88)	27.30(3.23)	4.80(3.09)	< 0.001	
Health Knowledge & Behavior					< 0.001
Intervention group (n = 51)	44.06(15.02)	56.58(9.79)	12.52(9.27)	< 0.001	
Control group (n = 49)	43.98(15.08)	49.92(12.37)	5.94(8.12)	< 0.001	
Perceived Health					0.552
Intervention group (n = 51)	11.86(1.80)	18.02(0.74)	6.16(1.91)	< 0.001	
Control group (n = 49)	11.82(1.84)	17.74(1.03)	5.92(2.11)	< 0.001	
Total					0.018
Intervention group (n = 51)	204.36(44.02)	271.46(21.26)	67.10(25.52)	< 0.001	
Control group (n = 49)	204.06(45.24)	257.42(27.84)	53.36(31.24)	< 0.001	

### Quality of life

The results of quality-of-life scores are presented in Table 3. The physical activity, social conditions, emotional health, functional status, additional attention, and total scores were significantly improved after the intervention (*P* < 0.05). Moreover, we noted greater changes in social conditions (1.70 ± 1.16 vs. 0.62 ± 0.83; *P* < 0.001), emotional health (4.02 ± 1.81 vs. 1.22 ± 1.30; *P* < 0.001), functional status (2.12 ± 1.56 vs. 0.42 ± 0.73; *P* < 0.001), additional attention (2.06 ± 1.57 vs. 0.82 ± 2.13; *P* = 0.001), and total scores (12.80 ± 5.28 vs. 5.78 ± 3.58; *P* < 0.001) in the intervention group compared with the control group. However, the change in physical activity between groups was not statistically significant (2.90 ± 1.20 vs. 2.70 ± 1.15; *P* = 0.396).

**Table 3:** Comparison of Quality of Life Scores

Items	Before intervention	After intervention	Mean difference	***P*** value for mean change	***P*** value between group
Physical activity					0.396
Intervention group (n = 51)	14.02(1.92)	16.92(1.55)	2.90(1.20)	< 0.001	
Control group (n = 49)	14.06(1.91)	16.76(1.53)	2.70(1.15)	< 0.001	
Social conditions					< 0.001
Intervention group (n = 51)	15.28(2.23)	16.98(1.60)	1.70(1.16)	< 0.001	
Control group (n = 49)	15.20(2.21)	15.82(1.96)	0.62(0.83)	< 0.001	
Emotional state					< 0.001
Intervention group (n = 51)	11.84(1.93)	15.86(1.07)	4.02(1.81)	< 0.001	
Control group (n = 49)	11.86(1.84)	13.08(1.16)	1.22(1.30)	< 0.001	
Functional status					< 0.001
Intervention group (n = 51)	14.82(2.38)	16.94(1.98)	2.12(1.56)	< 0.001	
Control group (n = 49)	14.78(2.26)	15.20(2.01)	0.42(0.73)	< 0.001	
Additional attention					0.001
Intervention group (n = 51)	13.04(1.92)	15.10(1.40)	2.06(1.57)	< 0.001	
Control group (n = 49)	12.88(2.61)	13.70(1.61)	0.82(2.13)	0.009	
Total					< 0.001
Intervention group (n = 51)	69.00(9.70)	81.80(5.91)	12.80(5.28)	< 0.001	
Control group (n = 49)	68.78(9.50)	74.56(7.23)	5.78(3.58)	< 0.001	

### Self-care Agency Scale

Table 4 shows the Self-Care Agency Scale scores. General self-care skills, developed self-care skills, self-care skills with poor health status, and total scores were significantly higher after intervention in both groups (*P* < 0.001). Moreover, we noted greater changes in general self-care skills (4.32 ± 5.87 vs. 2.40 ± 0.62; *P* = 0.016), developed self-care skills (3.70 ± 2.41 vs. 2.47 ± 0.71; *P* < 0.001), and total scores (10.84 ± 7.81 vs. 7.47 ± 1.51; *P* = 0.002) in the intervention group than in the control group. However, no significant difference between groups regarding changes in self-care skills with poor health status was observed (2.82 ± 1.60 vs. 2.60 ± 0.98; *P* = 0.362).

**Table 4:** Comparison of self-care agency scale scores

Items	Before intervention	After intervention	Mean difference	***P*** value for mean change	***P*** value between group
General self-care skill					0.016
Intervention group (n = 51)	20.19(3.99)	24.51(4.31)	4.32(5.87)	< 0.001	
Control group (n = 49)	20.21(4.04)	22.61(3.82)	2.40(0.62)	< 0.001	
Developed self-care skill					< 0.001
Intervention group (n = 51)	13.70(1.46)	17.40(1.89)	3.70(2.41)	< 0.001	
Control group (n = 49)	13.75(1.62)	16.23(1.23)	2.47(0.71)	< 0.001	
Self-care skill at poor health status					0.362
Intervention group (n = 51)	19.40(3.70)	22.23(3.26)	2.82(1.60)	< 0.001	
Control group (n = 49)	19.46(3.69)	22.05(3.28)	2.60(0.98)	< 0.001	
Total					0.002
Intervention group (n = 51)	53.30(8.74)	64.14(6.14)	10.84(7.81)	< 0.001	
Control group (n = 49)	53.42(8.89)	60.89(7.78)	7.47(1.51)	< 0.001	

## Discussion

This study aimed to assess the effects of nurse-led telephone follow-up according to the revised NOC for nursing outcomes, quality of life, and self-care abilities of patients after laryngectomy. A total of 100 patients with diverse characteristics were recruited. This study found greater improvements in psychosocial health, health knowledge and behavior, and total scores for nursing outcomes for patients in the intervention group. Moreover, patients in the intervention group saw significant improvements in social conditions, emotional health, functional status, additional attention, and total scores of quality of life than those in the control group. Furthermore, compared with traditional telephone follow-up, telephone follow-up conducted according to the revised NOC significantly improved general self-care skills, developed self-care skills, and total scores on the Self-Care Agency Scale.

Our study found that patients in the intervention group experienced greater improvements in psychosocial health, health knowledge and behavior, and total scores for nursing outcomes. A previous study found that after laryngectomy, patients presented with voice loss and reduced physical function resulting from tracheostomy respiration, which caused long-term changes in daily and professional life [22]. Moreover, anxiety and depression scores increased, and levels of stress were high for patients with laryngeal carcinoma [23–27]. The revised NOC includes questions related to mental health, which could help patients improve their mental well-being; it may also help nurses provide health guidance to improve psychosocial health. Moreover, patients may encounter issues such as disease recurrence, interruption of treatment information, and a lack of health care knowledge after surgical discharge [28]. Telephone follow-up according to the revised NOC repeatedly reinforces information that can positively affect knowledge acquisition and behavior compliance. Finally, the nurse-led telephone follow-up was performed by otolaryngology clinical specialty nurses, and the effect of the nursing intervention could be directly evaluated.

We noted that compared with the control group, patients in the intervention group experienced greater improvements in social condition, emotional state, functional status, additional attention, and in total scores of quality of life. Previous studies revealed negative changes in quality of life for patients with laryngeal carcinoma within the first year after surgery [29]. Improvements in quality of life were observed in some patients after long-term follow-up [30]. The telephone follow-up used in this study found improvement in all quality-of-life items except physical activity. This could be because patients in both the intervention and control groups were more concerned about their physiological conditions. Moreover, there was a focus on physiological functions related to the health guidance provided by clinicians during the follow-up period.

Nurse-led telephone follow-up according to the revised NOC is associated with greater improvements in general self-care skills, developed self-care skills, and total scores on the Self-Care Agency Scale. Previous studies have found that the use of telephone follow-up provides effective communication between nurses and patients, increases patient compliance, and improves self-care abilities. Moreover, the telephone follow-up in this study, improved patients’ self-care abilities through continuous feedback and supervision, which established and maintained effective self-care behaviors. Finally, the health status of patients varies; thus, a variety of forms of follow-up can play an important role in improving patient health.

This study has the following limitations. (1) Patients in this study were recruited from a single center and the sample size was small; hence, the representativeness of the findings was restricted. (2) The nature of the study design meant that blinding of conditions could not be applied to the patients and nurses. (3) Disease status and background treatments were not assessed, and these may have played an important role in nursing outcomes, quality of life, and self-care abilities. (4) Stratified analyses were not performed. The results might have differed according to the patients’ characteristics. Finally, (5) the duration of follow-up was 6 months. Thus, future studies should evaluate the long-term effects of nurse-led telephone follow-up according to the revised NOC or traditional telephone follow-up for laryngeal carcinoma surgery patients.

## Conclusion

This study found that nurse-led telephone follow-up according to the revised NOC improved nursing outcomes (psychosocial health and health knowledge and behavior), quality of life (social conditions, emotional health, functional status, and additional attention), and self-care abilities (general self-care abilities and development self-care abilities) compared with traditional telephone follow-up for patients after laryngectomy. Further large-scale randomized controlled trials are warranted to verify the findings of this study and assess the long-term effects of nurse-led telephone follow-up according to the revised NOC.

## Electronic supplementary material

Below is the link to the electronic supplementary material.

Supplementary Material 1

Supplementary Material 2

Supplementary Material 3

Supplementary Material 4

## Data Availability

The datasets used and/or analyzed during the current study are available from the corresponding author on reasonable request.

## List of abbreviations

NOC: Nursing Outcomes Classification

## References

[1] Bray F, Ferlay J, Soerjomataram I, Siegel RL, Torre LA, Jemal A (2018). Global cancer statistics 2018: GLOBOCAN estimates of incidence and mortality worldwide for 36 cancers in 185 countries. CA Cancer J Clin.

[2] Zhang SS, Xia QM, Zheng RS, Chen WQ (2015). Laryngeal cancer incidence and mortality in China, 2010. J Cancer Res Ther.

[3] Li H, Wang Y, Zhu C, Wang X, Du L (2015). Incidence and mortality of laryngeal cancer in Zhejiang cancer registry, 2000–2011. J Cancer Res Ther.

[4] Bonhin RG, Rocha VB, de Carvalho GM, Guimarães AC, Crespo AN, Chone CT (2015). Correlation between vascular endothelial growth factor expression and presence of lymph node metastasis in advanced squamous cell carcinoma of the larynx. Braz J Otorhinolar.

[5] Petrakos I, Kontzoglou K, Nikolopoulos TP, Papadopoulos O, Kostakis A (2012). Glottic and supraglottic laryngeal cancer: epidemiology, treatment patterns and survival in 164 patients. J Buon.

[6] Laccourreye O, Malinvaud D, Menard M, Consoli S, Giraud P, Bonfils P (2014). Otorhinolaryngologists’ personal treatment preferences (total laryngectomy or laryngeal preservation) when faced with advanced stage laryngeal cancer. Eur Ann Otorhinolaryngol Head Neck Dis.

[7] Cox SR, Theurer JA, Spaulding SJ, Doyle PC (2015). The multidimensional impact of total laryngectomy on women. J Commun Disord.

[8] Townsley RB, Baring DE, Clark LJ (2014). Emergency department care of a patient after a total laryngectomy. Eur J Emerg Med.

[9] Nettleton J, Jelski J, Ahmad A (2020). Reducing readmissions and improving patient experience following urological surgery, through early telephone follow-up. BMJ Open Qual.

[10] Walijee H, Sood S, Markey A, Krishnan M, Lee A, De S (2020). Is nurse-led telephone follow-up for post-operative obstructive sleep apnoea patients effective? A prospective observational study at a paediatric tertiary centre. Int J Pediatr Otorhinolaryngol.

[11] Gülşen M, Akansel N (2020). Effects of Discharge Education and Telephone Follow-up on Cataract Patients’ Activities According to the Model of Living. J Perianesth Nurs.

[12] Vance S, Fontecilla N, Samie FH, Patel V, Lewin JM (2019). Effect of Postoperative Telephone Calls on Patient Satisfaction and Scar Satisfaction After Mohs Micrographic Surgery. Dermatol Surg.

[13] Thompson JC, Cichowski SB, Rogers RG, Qeadan F, Zambrano J, Wenzl C (2019). Outpatient visits versus telephone interviews for postoperative care: a randomized controlled trial. Int Urogynecol J.

[14] Moorhead S, Johnson M, Mass M, Swanson E (2012). Nursing outcomes classification (NOC).

[15] Moorhead S, Swanson E, Johnson M, Maas M (2018). Nursing outcomes classification (NOC): measurement of health outcomes.

[16] Ding YX, Yang H, Sun YX, Xu J, Jing L, Ning Y (2021). Evaluation and revision of core postoperative nursing outcomes for laryngeal carcinoma in China. BMC Nurs.

[17] Morato-Galán M, Caminero Cueva MJ, Rodrigo JP, Suárez Nieto C, Núñez-Batalla F (2014). Assessment of vocal quality following treatment of advanced pharyngo-laryngeal carcinoma with a protocol of organ preservation. Acta Otorrinolaringol Esp.

[18] Beisland E, Hauge EM, Aarstad AKH, Hjermstad J, Aarstad HJ, Beisland C (2020). Personality and educational level determine self-reported health-related quality-of-life and distress in patients with renal tumors awaiting radical surgery. Scand J Urol.

[19] Soderhamn O, Ek A, Porn I (1996). The self-care ability scale for the elderly. Scand J Occup Ther.

[20] Wierzbicka M, Napierała J (2017). Updated National Comprehensive Cancer Network guidelines for treatment of head and neck cancers 2010–2017. Otolaryngol Pol.

[21] Malmström M, Ivarsson B, Klefsgård R, Persson K, Jakobsson U, Johansson J (2016). The effect of a nurse led telephone supportive care programme on patients’ quality of life, received information and health care contacts after oesophageal cancer surgery-A six month RCT-follow-up study. Int J Nurs Stud.

[22] Cogwell AR, Anderson FK (2002). Psychological and psychosocial implications of head and neck cancer. Internet J Ment Health.

[23] Danker H, Wollbrück D, Singer S, Fuchs M, Brähler E, Meyer A (2010). Social withdrawal after laryngectomy. Eur Arch Otorhinolaryngol.

[24] de Maddalena H (2002). The influence of early speech rehabilitation with voice prostheses on the psychological state of laryngectomized patients. Eur Arch Otorhinolaryngol.

[25] Syse A, Tretli S, Kravdal Ø (2008). Cancer’s impact on employment and earnings–a population-based study from Norway. J Cancer Surviv.

[26] Singer S, Danker H, Dietz A, Hornemann B, Koscielny S, Oeken J (2008). Screening for mental disorders in laryngeal cancer patients: a comparison of 6 methods. Psychooncology.

[27] Kugaya A, Akechi T, Okuyama T, Nakano T, Mikami I, Okamura H (2000). Prevalence, predictive factors, and screening for psychologic distress in patients with newly diagnosed head and neck cancer. Cancer.

[28] Mannelli G, Santoro R, Segala F, Surrenti E, Gallo O (2018). Gastro-pharyngeal reflux and total laryngectomy. Increasing knowledge about its management. Am J Otolaryngol.

[29] Hammerlid E, Mercke C, Sullivan M, Westin T (1998). A prospective quality of life study of patients with laryngeal carcinoma by tumor stage and different radiation therapy schedules. Laryngoscope.

[30] Hammerlid E, Silander E, Hörnestam L, Sullivan M. Health-related quality of life three years after diagnosis of head and neck cancer–a longitudinal study. Head Neck. 2001;23(2):113–25. doi: https://doi.org/10.1002/1097-0347(200102)23:2<113::aid-hed1006>3.0.co;2-w. 10.1002/1097-0347(200102)23:2<113::aid-hed1006>3.0.co;2-w 11303628

