# Supplementary material for: Nurse-led telephone follow-up according to the revised nursing outcomes classification for laryngeal carcinoma surgery patients: a randomized controlled trial
Source: BMC Nurs. 2022 Oct 17;21:281. doi: 10.1186/s12912-022-01054-2 (PMC9578269; doi:10.1186/s12912-022-01054-2)
Supplement: Supplementary file 1 — Supplementary Material 1 [file 12912_2022_1054_MOESM1_ESM.doc]

**Nursing outcome after laryngectomy**

Name: Admission No: Age: Sex:

Disease status: Surgery type: Admission date:

Evaluation of time: Evaluation nurse:

| 0802 Vital Signs Total score | | | | | |
| --- | --- | --- | --- | --- | --- |
| Score/index | Extreme deviation | Severe deviation | Moderate deviation | Slight deviation | Normal |
|  | 1 | 2 | 3 | 4 | 5 |
| 080201 Body temperature |  |  |  |  |  |
| 080203 Pulse |  |  |  |  |  |
| 080204 Respiration |  |  |  |  |  |
| 080205 Systolic blood pressure |  |  |  |  |  |
| 080206 Disatolic blood pressure |  |  |  |  |  |
| 0415 Respiratory Status Total score | | | | | |
| Score/index | Extreme deviation | Severe deviation | Moderate deviation | Slight deviation | Normal |
|  | 1 | 2 | 3 | 4 | 5 |
| 041501 Respiratory rate |  |  |  |  |  |
| 041502 Respiratory rhythm |  |  |  |  |  |
| 041532 Airway patency |  |  |  |  |  |
| 040208 PaO2 |  |  |  |  |  |
| 040209 PaCO2 |  |  |  |  |  |
| 041514 Dyspnea at rest |  |  |  |  |  |
| 040309 Accessory muscle use |  |  |  |  |  |
| 040331 Accumulation of sputum |  |  |  |  |  |
| 0703 Infection Severity Total score | | | | | |
| Score/index | Extreme deviation | Severe deviation | Moderate deviation | Slight deviation | Normal |
|  | 1 | 2 | 3 | 4 | 5 |
| 070303 Full-smelling discharge |  |  |  |  |  |
| 070304 Purulent sputum |  |  |  |  |  |
| 070305 Purulent drainage |  |  |  |  |  |
| 070334 Tenderness |  |  |  |  |  |
| 070319 Chest X-ray infiltration |  |  |  |  |  |
| 070326 White blood count elevation |  |  |  |  |  |
| 0902 Communication Total score | | | | | |
| Score/index | Extreme deviation | Severe deviation | Moderate deviation | Slight deviation | Normal |
|  | 1 | 2 | 3 | 4 | 5 |
| 090201 Use written language |  |  |  |  |  |
| 090202 Use of spoken language |  |  |  |  |  |
| 040330 Impaired vocalization |  |  |  |  |  |
| 101302 Changes in voice quality |  |  |  |  |  |
| 1013 Swallowing Condition: Pharyngeal Phase Total score | | | | | |
| Score/index | Extreme deviation | Severe deviation | Moderate deviation | Slight deviation | Normal |
|  | 1 | 2 | 3 | 4 | 5 |
| 101304 Number of swallows appropriate for bolus size |  |  |  |  |  |
| 101303 Choking |  |  |  |  |  |
| 101314 Coughing |  |  |  |  |  |
| 101315 Gagging |  |  |  |  |  |
| 101306 Increased swallow effort |  |  |  |  |  |
| 101310 Nasal reflux |  |  |  |  |  |
| 101316 Aspirations |  |  |  |  |  |
| 1004 Nutritional Status Total score | | | | | |
| Score/index | Extreme deviation | Severe deviation | Moderate deviation | Slight deviation | Normal |
|  | 1 | 2 | 3 | 4 | 5 |
| 100401 Nutrient intake |  |  |  |  |  |
| 100405 Weight/height ratio |  |  |  |  |  |
| 1101 Tissue Integrity: Skin&Mucous Membrane Total score | | | | | |
| Score/index | Extreme deviation | Severe deviation | Moderate deviation | Slight deviation | Normal |
|  | 1 | 2 | 3 | 4 | 5 |
| 110101 Skin temperature |  |  |  |  |  |
| 110102 Sensation |  |  |  |  |  |
| 110113 Skin interity |  |  |  |  |  |
| 110117 Scar tissue |  |  |  |  |  |
| 110124 Induration |  |  |  |  |  |
| 110125 Corneal abrasion |  |  |  |  |  |
| 1300 Acceptance: Health Status Total score | | | | | |
| Score/index | Extreme deviation | Severe deviation | Moderate deviation | Slight deviation | Normal |
|  | 1 | 2 | 3 | 4 | 5 |
| 13008 Recognizes reality of health situation |  |  |  |  |  |
| 130009 Pursues information about health |  |  |  |  |  |
| 130010 Copes with health situation |  |  |  |  |  |
| 130014 Performs self-care tasks |  |  |  |  |  |
| 1308 Adaptation to Physical Disabilities | | | | | |
| Score/index | Extreme deviation | Severe deviation | Moderate deviation | Slight deviation | Normal |
|  | 1 | 2 | 3 | 4 | 5 |
| 130803 Adapts to functional limitations |  |  |  |  |  |
| 1206 Will to Live Total score | | | | | |
| Score/index | Extreme deviation | Severe deviation | Moderate deviation | Slight deviation | Normal |
|  | 1 |  | 3 | 4 | 5 |
| 120602 Expression of hope |  |  |  |  |  |
| 120603 Expression of optimism |  |  |  |  |  |
| 120613 Use of treatments to lengthen life |  |  |  |  |  |
| 120609 Use of strategies to enhance health |  |  |  |  |  |
| 1842 Knowledge: Infection Management Total score | | | | | |
| Score/index | Extreme deviation | Severe deviation | Moderate deviation | Slight deviation | Normal |
|  | 1 | 2 | 3 | 4 | 5 |
| 184204 Signs and symptoms of infection |  |  |  |  |  |
| 184207 Iimportance of hand sanitation |  |  |  |  |  |
| 184209 Treatment for diagnosed infection |  |  |  |  |  |
| 184219 Risk of drug resistance |  |  |  |  |  |
| 1625 Smoking Cessation Behavior Total score | | | | | |
| Score/index | Extreme deviation | Severe deviation | Moderate deviation | Slight deviation | Normal |
|  | 1 | 2 | 3 | 4 | 5 |
| 162502 Express belief in the ability to stop smoking |  |  |  |  |  |
| 162504 Identifies negative consequences of tobacoo use |  |  |  |  |  |
| 162505 Develop effective strategies to eliminate tobacco use |  |  |  |  |  |
| 162523 Use available support groups |  |  |  |  |  |
| 1918 Aspiration Preventing Total score | | | | | |
| Score/index | Extreme deviation | Severe deviation | Moderate deviation | Slight deviation | Normal |
|  | 1 | 2 | 3 | 4 | 5 |
| 191801 Identify risk factors |  |  |  |  |  |
| 191802 Avoid risk factors |  |  |  |  |  |
| 191803 Positions self upright for eadting and drinking |  |  |  |  |  |
| 191804 Selects foods according to swallowing ability |  |  |  |  |  |
| 191805 Positions self on side for eating and drinking as need |  |  |  |  |  |
| 191808 Uses liquid thickeners as needed |  |  |  |  |  |
| 191809 Maintains oral hygiene |  |  |  |  |  |
| 191810 Remains upright for 30 minutes after eating |  |  |  |  |  |
| 2102 Pain Level Total score | | | | | |
| Score/index | Extreme deviation | Severe deviation | Moderate deviation | Slight deviation | Normal |
|  | 1 | 2 | 3 | 4 | 5 |
| 21024 Length of pain episodes |  |  |  |  |  |
| 210221 Rubbing affected area |  |  |  |  |  |
| 210206 Facial expressions of pain |  |  |  |  |  |
| 210225 Tearing |  |  |  |  |  |
