# Supplementary material for: Nurse-led telephone follow-up according to the revised nursing outcomes classification for laryngeal carcinoma surgery patients: a randomized controlled trial
Source: BMC Nurs. 2022 Oct 17;21:281. doi: 10.1186/s12912-022-01054-2 (PMC9578269; doi:10.1186/s12912-022-01054-2)
Supplement: Supplementary file 2 — Supplementary Material 2 [file 12912_2022_1054_MOESM2_ESM.doc]

**The Chinese version of FACT-H&N**

| Physical activity | Never (0) | Few (1) | Sometimes (2) | More (3) | Plenty (4) |
| --- | --- | --- | --- | --- | --- |
| 1.I'm feeling low on energy |  |  |  |  |  |
| 2.I fell sick |  |  |  |  |  |
| 3.I can't meet the needs of my family because of my health |  |  |  |  |  |
| 4.I fell pain |  |  |  |  |  |
| 5.The side effects of the treatment made me feel sick |  |  |  |  |  |
| 6.I'm not feeling well |  |  |  |  |  |
| 7.I was forced to stay in bed |  |  |  |  |  |
| Social conditions |  |  |  |  |  |
| 8.I feel close to my friends |  |  |  |  |  |
| 9.I get emotional support from my family |  |  |  |  |  |
| 10.I have the support of my friends |  |  |  |  |  |
| 11.My family has accepted my illness |  |  |  |  |  |
| 12.I was pleased with the communication with my family about my illness |  |  |  |  |  |
| 13. I'm happy with my sex life (if you wouldn't)  Please ignore the answer. |  |  |  |  |  |
| 14.I feel close to my lover |  |  |  |  |  |
| Emotional state |  |  |  |  |  |
| 15.I feel sad |  |  |  |  |  |
| 16.I'm happy with the way I'm fighting the disease |  |  |  |  |  |
| 17.I lost faith in conquering the disease |  |  |  |  |  |
| 18.I feel nervous |  |  |  |  |  |
| 19.I'm afraid I might die |  |  |  |  |  |
| 20.I feared I would get worse |  |  |  |  |  |
| Functional status |  |  |  |  |  |
| 21.I am able to work (including working from home) |  |  |  |  |  |
| 22.My work, including at home, gives me a sense of accomplishment |  |  |  |  |  |
| 23.I can enjoy life |  |  |  |  |  |
| 24.I have been able to face my illness |  |  |  |  |  |
| 25.I slept very well |  |  |  |  |  |
| 26.I'm enjoying the entertainment I used to do |  |  |  |  |  |
| 27.I am satisfied with the quality of my life now |  |  |  |  |  |
| Additional attention |  |  |  |  |  |
| 28.I can eat the food I like |  |  |  |  |  |
| 29.My mouth is dry and my tongue is dry |  |  |  |  |  |
| 30.I have difficulty breathing |  |  |  |  |  |
| 31.My voice has the usual timbre and power |  |  |  |  |  |
| 32.I can eat as much food as I want |  |  |  |  |  |
| 33.I was uncomfortable with the appearance of my face and neck |  |  |  |  |  |
| 34.I can swallow easily |  |  |  |  |  |
| 35.I smoke cigarettes or other tobacco products |  |  |  |  |  |
| 36. I drink (eg beer, wine, etc) |  |  |  |  |  |
| 37.I can communicate with other people |  |  |  |  |  |
| 38.I can eat solid food |  |  |  |  |  |
