# Supplementary material for: Nurse-led telephone follow-up according to the revised nursing outcomes classification for laryngeal carcinoma surgery patients: a randomized controlled trial
Source: BMC Nurs. 2022 Oct 17;21:281. doi: 10.1186/s12912-022-01054-2 (PMC9578269; doi:10.1186/s12912-022-01054-2)
Supplement: Supplementary file 3 — Supplementary Material 3 [file 12912_2022_1054_MOESM3_ESM.doc]

**The Chinese version of the Appraisal of Self-Care Agency Scale**

|  | Strongly disagree | Disagree | Uncertain | Agree | Complete agree |
| --- | --- | --- | --- | --- | --- |
| 1.When the environment changes, I will make necessary adjustments to maintain my health |  |  |  |  |  |
| 2.If my performance declines, I will make necessary adjustments |  |  |  |  |  |
| 3.When necessary, I will take the best way to keep healthy |  |  |  |  |  |
| 4.I know what I need to do to take care of myself, but I often lack energy |  |  |  |  |  |
| 5.I will look for better ways to take care of myself |  |  |  |  |  |
| 6.I take time to take care of myself when necessary |  |  |  |  |  |
| 7.If I take a new drug, I will understand the side effects so that I can take better care of myself |  |  |  |  |  |
| 8.I used to change some old habits to improve my health |  |  |  |  |  |
| 9.I usually take steps to keep myself and my family healthy |  |  |  |  |  |
| 10.I constantly evaluate the effectiveness of what I do for my health |  |  |  |  |  |
| 11.In my daily activities, I seldom spend time taking care of myself |  |  |  |  |  |
| 12.When my health is threatened, I can get the information I want |  |  |  |  |  |
| 13.When I can't take care of myself, I ask for help |  |  |  |  |  |
| 14.I rarely take time for myself |  |  |  |  |  |
| 15.I can't always take care of myself the way I like |  |  |  |  |  |
