# Supplementary material for: Nurse-led telephone follow-up according to the revised nursing outcomes classification for laryngeal carcinoma surgery patients: a randomized controlled trial
Source: BMC Nurs. 2022 Oct 17;21:281. doi: 10.1186/s12912-022-01054-2 (PMC9578269; doi:10.1186/s12912-022-01054-2)
Supplement: Supplementary file 4 — Supplementary Material 4 [file 12912_2022_1054_MOESM4_ESM.doc]

**Nurse-led telephone follow-up sheet**

Name: Admission No: Age: Sex:

Disease status: Surgery type: Admission date:

| Flowchart | Content | Outcome | Guide |
| --- | --- | --- | --- |
| Connect | Hello, excuse me.  May I ask if you are the ** Mrs/Mr ** who was hospitalized in ** Hospital? | The degree of voice  Changes in sound quality  The degree to which oral language is used |  |
| Introduce | I am a nurse in the Department of Otolaryngology, Head and Neck of XXX Hospital.  Now I call you mainly to follow up your health condition after you are discharged from the hospital and answer some related questions. |  |  |
| Communicate | Would that be convenient for you?  The whole process may take about 30 minutes.  Yes: OK, please be ready for follow-up. If you have difficulty expressing or feel tired, you can ask your family members for assistance.  No: I'm sorry to bother you.  When will it be convenient for you to call us again? Is tomorrow (a specific day within a week) OK?  Patient has passed away: I am sorry to hear that.  Could you tell me when and why patient died?  Thank you so much! |  |  |
| Ask and respond | 1.When was the last time you went to the hospital for review? |  |  |
|  | 2.Is your temperature normal recently? |  |  |
|  | 3.What is your blood pressure and pulse now? |  |  |
|  | 4.How smooth is your breathing? |  |  |
|  | 5.What is your breathing rate? |  |  |
|  | 6.How much difficulty do you have in breathing |  |  |
|  | 7.How much do you use a ventilator at home? |  |  |
|  | 8.How well do you cough up your sputum? |  |  |
|  | 9.How about your sputum? |  |  |
|  | 10.What is the level of rancid discharge from your throat |  |  |
|  | 11.Do you have any pain in your chest? How severe is it? |  |  |
|  | 12.Have you had a chest X-ray in the last week?  If so, what were the results? |  |  |
|  | 13.Have you had a blood test in the last week?  If so, what were the results? |  |  |
|  | 14.How hard do you feel swallowing? |  |  |
|  | 15.How often do you cough when you swallow? |  |  |
|  | 16.Do you have any vomiting when you eat or swallow? |  |  |
|  | 17.Do you have any nasal reflux when you swallow or eat? |  |  |
|  | 18.Have you inhaled food while swallowing? |  |  |
|  | 19.Do you think the number of times you swallow corresponds to the size and texture of your food?  Do you feel like you have to swallow big food in one go or small food that takes multiple times to get in? |  |  |
|  | 20.How much do you weigh now? |  |  |
|  | 21.How many staples, vegetables, meat and eggs do you eat in your daily diet? |  |  |
|  | 22.Is the temperature of your skin normal at the wound?  (Selecting body temperature according to operation method) |  |  |
|  | 23.Does the skin on your wound feel normal?  (Selecting body temperature according to operation method) |  |  |
|  | 24.Do you have any scars on your wound?  (Selecting body temperature according to operation method) |  |  |
|  | 25.What is the degree of skin necrosis at your wound?  (Selecting body temperature according to operation method) |  |  |
|  | 26.How about the degree of induration on your wound?  (Selecting body temperature according to operation method) |  |  |
|  | 27.How comfortable are you with your current health? |  |  |
|  | 28.How are you adjusting to your current functional state? |  |  |
|  | 29.What steps do you take to deal with your health? |  |  |
|  | 30.How much hope do you have for your health? |  |  |
|  | 31.How optimistic are you about your health? |  |  |
|  | 32.To what extent are you actively taking care of your health? |  |  |
|  | 33.To what extent do you actively seek out information related to your health? |  |  |
|  | 34.How do you use strategies to improve your health? |  |  |
|  | 35.Do you know the signs and symptoms of infection? |  |  |
|  | 36.Do you know of any treatments for infection? |  |  |
|  | 37.Do you know how to do good hand hygiene? |  |  |
|  | 38.Do you know how antibiotic resistance affects infection? |  |  |
|  | 39.Do you smoke at present? (If yes, please answer questions 40-47.) |  |  |
|  | 40.How do you feel about your confidence to quit smoking? |  |  |
|  | 41.How much do you know about the consequences of smoking? |  |  |
|  | 42.What strategies have you developed for yourself to quit smoking? |  |  |
|  | 43.Do you use substitutes instead of smoking? |  |  |
|  | 44.How well do you control the risk factors of smoking?  For example, stay away from friends who smoke and post warnings in the room |  |  |
|  | 45.What do you know about the risk factors for aspiration? |  |  |
|  | 46.Do you know how to avoid the risk factors of aspiration? |  |  |
|  | 47.How often do you eat or drink in an upright position |  |  |
|  | 1. How well do you maintain an upright position for 30 minutes after eating? |  |  |
|  | 49.How often do you lie on your side when you need to eat or drink? |  |  |
|  | 50. Do you usually choose food based on your swallowing ability? |  |  |
|  | 51.To what extent do you use a liquid thickener when necessary? |  |  |
|  | 52.How well do you keep your mouth clean? |  |  |
|  | 53.What is the duration of your pain episodes? |  |  |
|  | 54.How much tenderness do you feel about the wound? |  |  |
|  | 55.How do you experience tearing pain? |  |  |
|  | 56.What is your own facial expression when you feel pain?  (Please help to answer) |  |  |
| Exit | Thank you very much for your detailed answer. Have we answered your questions?  Yes: thank you for your support and cooperation, look forward to your regular visit.  Our next follow-up visit will be held around * *, and we will contact you 2 days in advance.  Wish you a happy life!  No: What can I do for you? |  |  |
| Summarized | Time (points): |  |  |
|  | Physiologic health |  |  |
|  | Psychosocial health |  |  |
|  | Health knowledge & behavior |  |  |
|  | Perceived health |  |  |
|  | The most important problems of patients: |  |  |
|  | Problems that the visitors failed to solve: |  |  |
|  | Expected solution: |  |  |

Evaluation of time: Evaluation nurse:
